# The Sustainability of Seafood Products in the Opinions of Italian Consumers of Generation Z

**DOI:** 10.3390/foods12224047

**Published:** 2023-11-07

**Authors:** Maria Bonaventura Forleo, Marilena Bredice

**Affiliations:** Department of Economics, University of Molise, 86100 Campobasso, Italy; m.bredice@studenti.unimol.it

**Keywords:** marine ecosystem services, food provisioning value, environmental consciousness, seafood consumption, socio-demographics, cluster analysis

## Abstract

This study aims to explore whether members of Generation Z have sensitivity and awareness about environmental issues related to seafood production and consumption, their beliefs on how to make more sustainable the future provisioning of seafood, their consumption frequency, and, finally, whether different profiles and groups of people could be detected. A survey was implemented with 778 Italian students attending secondary schools. Descriptive statistics, testing, and cluster analyses were applied. Results provide the sustainability profile of five groups, of which three are aligned with SDGs 12 and 14, but the other groups, comprising almost half of the sample, are insensitive, unaware, or irresolute about the sustainability of seafood production and consumption. Overall, people’s environmental consciousness does not appear to be strongly related to the frequency of consumption of sustainable seafood species. Regarding the solutions for improving the sustainability of future seafood production, young respondents underlined the catching and raising of novel, discarded, not exploited, or marginally exploited seafood species. People declared a high knowledge of the nutritional and safety implications of seafood. This study is one of the few that explore issues specifically related to the profiles of Generation Z and young people’s approach toward sustainable seafood production and consumption.

## 1. Introduction

The sustainability of food consumption is a worldwide-spread topic addressed at the political level by the United Nations’ 2030 Agenda for Sustainable Development [1] and especially by the Sustainable Development Goal (SDG) 12 aimed at fostering an integrated approach toward responsible consumption and production patterns. As regards seafood consumption, the SDG 14 should be also mentioned, devoted to conservation and sustainably using the oceans, seas, and marine resources.

The fisheries and aquaculture sectors significantly contribute to food security and nutrition, ensuring the livelihoods of people around the world. Fish production and consumption have increased dramatically over the past few years, exceeding 20 kilos per capita per year globally, with a minimum of about 10 kilos in Africa and levels of 21 and 25 kilos in Europe and Asia [2]. However, nowadays, more than 90% of the world’s fish stocks are heavily overexploited, depleted, or collapsed [3].

Although there are several challenges facing the food system [4], the availability of food is not perceived as an immediate, major concern [5] but urgency is required for preserving ocean food production systems for global future food and nutritional security [6]. Recommended actions involve increasing the awareness, accountability, and stewardship of producers and consumers in order to better inform their choices [7].

For a more sustainable future seafood system, special importance should be given to the young generations that will dominate the food demand side in the medium and long term. Indeed, global trends highlight the growing interest of young people in sustainability issues. As concerns the awareness and sensitivity of young generations, the 2022 Deloitte Report affirms that Millennials and Gen Zs (consisting of individuals born between 1981–1996 and in or after 1997, respectively [8]) are doing their part making efforts to protect the environment, and that younger people place environmental consciousness among their top personal concerns. In-depth knowledge of sustainable food consumption opinions and the behavior of today’s younger generations can reveal future demand prospects that could support an improvement in the environmental impact of seafood systems.

Despite much research on sustainable food consumption among young people, the theme of seafood consumption among Generation Z in terms of awareness, concerns, opinions, and purchasing choices still remains scarce. Furthermore, little scientific evidence has analyzed Gen Z’s opinions on future scenarios of food production from the sea. By analyzing Gen Z’s beliefs toward sustainable seafood consumption, research can provide valuable insights to understand Gen Z’s consumption habits, preferences, and concerns regarding seafood, and to envisage future scenarios of demand for food from the sea.

In this context, this study investigates the youth of Generation Z with the following aims: (1) to classify young people’s profiles based on their sensitivity, concerns, and beliefs regarding the sustainability of seafood supply and demand; (2) to describe youth segments based on their seafood consumption, personal characteristics, and territorial backgrounds; and (3) to evaluate which segments, if any, are likely to respond positively to sustainable seafood consumption.

## 2. Literature Background

### 2.1. Young Generations toward Sustainable Seafood

For a first definition of sustainable consumption one can refer to the Oslo Symposium on Sustainable Consumption held in 1994, which defined it as “the use of goods and services that respond to basic needs and bring a better quality of life, while minimizing the use of natural resources, toxic materials and emissions of waste and pollutants over the life cycle, so as not to jeopardize the needs of future generations” [9,10].

In particular, sustainable seafood consumption has been defined as the consumption of “seafood caught or raised in a way that maintains or increases production over the long term, without compromising the health or function of the web of life in our oceans” [11]. The literature about environmentally sustainable seafood consumption is quite rich and grows at an exponential rate, dealing with a great variety of topics. Many studies have focused on how the definition of sustainable fish consumption can be transformed into concrete actions, identifying milestones to promote this behavior [12]. Consumers’ attitudes, awareness, preferences, and behaviors as well as factors influencing them have been investigated in many studies [13,14,15,16,17,18,19,20,21]. Other researchers have analyzed the differences between consumers with greater and lesser knowledge of seafood products, showing that the former is more oriented toward sustainable purchasing choices and more diversified consumption of seafood species [22,23]. Other studies have investigated future scenarios [24] and suggested research and policy agenda for improving the sustainability of seafood consumption [25]. Finally, some studies have dealt with specific environmentally related topics such as the consumption of seafood byproducts [26], consumers’ preferences related to product variety and alternative food networks [27], and the relevance of eco-labels [28,29]; in addition, the ecological and carbon footprints of scenarios with different seafood consumption patterns have also been analyzed [30,31,32].

As for seafood consumption, there is limited knowledge about how large consumer groups with significant purchasing power, such as Millennials and Generation Z, perceive and interact with seafood. Indeed, it is important to carry out research on seafood sustainability among younger generations, considering that they represent the largest living group, dominate the labor force, and now possess the greatest purchasing power compared to any other generation [33].

Studies on food purchasing and consumption behavior among different generations indicate that young people exhibit distinct behaviors from older generations [34,35]. Sánchez et al. [34] reviewed studies about the sustainable food consumption behavior of university students and proposed a categorization of distinct behaviors but they did not consider seafood consumption.

Güney and Sangün [35] compared factors influencing seafood consumption among different generations in Turkey but they did not consider sustainable drivers.

Anuar et al. [36] analyzed the level of awareness of sustainable seafood products, purchasing behavior, and factors influencing the purchase of sustainable seafood products among Malaysian Millennials. The results revealed that most respondents are aware of sustainable seafood consumption. When purchasing seafood, Generation Y considers various aspects such as price, sustainability of seafood, its origin, and fishing methods; in addition, factors such as product labeling, recognizable logos, and more accessible green options could make it easier for consumers to practice sustainable seafood consumption.

The study by Gibson et al. [37] analyzed experiences of U.S. undergraduate students with seafood and how they perceived the role of seafood in nourishing people while sustaining the natural environment. The research emphasizes the interconnectedness of place attachment and family identity with consumption patterns. Key themes identified in relation to the significance of seafood in individuals’ diets encompass sustainability, regulations, restricted seafood consumption, and limited knowledge. These findings highlighted the emergence of Generation Z as a cohort that prioritizes sustainability.

Davis [38] studied the responses of consumers in New England toward underutilized fish species and discovered that younger generations showed interest in engaging in their consumption and wanted to share their experiences of fish consumption with friends and family.

Su et al. [39] classified U.S. Gen Zs into segments based on their environmental consciousness and assessed the relationships with their degree of ecological awareness, the importance of sustainable food attributes, their food choices, and socio-demographics. The authors argued that Generation Z consumers with high environmental consciousness and moderate ecological awareness strongly considered the ecological and health attributes of the product in their consumption choices, while Generation Z consumers with low environmental consciousness considered more the extrinsic product attributes, such as price and convenience.

Based on the above literature, two research questions were set as follows:

RQ.1. What does Gen Z believe about the future sustainability of seafood production and consumption? And what about seafood consumption habits?

RQ.2. Can Gen Z consumers be segmented into distinct groups based on their level of environmental awareness and opinions about sustainable fish supply and demand?

### 2.2. Socioeconomic Drivers of Gen Z Sustainable Food Consumption

Dealing with the socio-demographic profiles of “green fish consumers”, several reviews [14,15,16,35] reported a great heterogeneity of results in terms of gender, age, education, and income.

In addition, the study by Gibson et al. [37] suggested that geographic location is part of the experience of U.S. Gen Z with seafood, which implied that place attachment was intertwined with consumption behavior. The study by Su et al. [39] identified segments of the U.S. sustainable food market that differed by socio-demographic characteristics and found significant differences for the main effects of gender and region of residence.

Based on the above literature, a third research question was set as follows:

RQ.3. Do Gen Z segments differ with respect to socio-demographics regarding gender, place of residence, and frequency of consumption?

To answer the above research questions, this study investigates Italians of Generation Z in terms of their environmental consciousness about seafood production and consumption, with the aim of highlighting whether it is possible to identify different groups. In particular, based on a segmentation analysis, this study explores whether Generation Z has sensitivity and awareness about environmental issues related to the exploitation of marine resources for human consumption, their beliefs on how to make more sustainable the future provisioning of seafood for the market, and Gen Z’s seafood consumption choices.

## 3. Materials and Methods

### 3.1. Gathering Data

A direct survey was implemented with students in four cities located in central Italy (Abruzzo and Molise regions) between January and April 2022. Cities differ by size (2 of medium and 2 of small size) and location (2 along the coast, 2 in the immediate inner areas). The sample of respondents was homogeneous by age, being formed by students attending the final year’s class of secondary high school, aged 18–20 years. The sampling method was based on a two-stage strategy: at first, all secondary schools in each city were considered and invited to take part in the activities; the second-stage units, i.e., all willing classes and students in each school, were met and involved in the study. After a brief presentation of the study aims, an anonymous questionnaire was administered using an online platform. Students were invited to give their consent and received the required privacy information according to the EU General Data Protection Regulation [40].

The questionnaire was structured in several sections that are described below.

In a first section, two questions investigated students’ environmental consciousness —in terms of sensitivity, awareness, and concern—by adapting to the context of the marine environment the approach proposed by Sánchez et al. [34], First, people’s sensitivity about the environmental status of the marine ecosystem was considered (sensitivity). In a second question, students’ general awareness and concern about the existence of potential conflicts between marine resources conservation goals and economic activities was measured (marine protection conflicts). Sensitivity and awareness were measured on a 5-point Likert scale as in [34], from “Strongly disagree” (1 point) to “Strongly agree” (5 points).

The second section investigated opinions about the value of ecosystem services and related scenarios in terms of food provisioning.

A first sphere, composed of two questions, dealt with the values of marine ecosystems that students considered relevant within the next ten years. Two perspectives were approached: in a first question, the most important values of the marine environment and resources were investigated (ME_importance_; from 1 = ”very unimportant”, to 5 = ”very important”); a second question dealt with the values most threatened by fishing and farming activities (ME_menace_; from 1 = ”not at all menaced”, to 5 = ”the most menaced”); the 5-point Likert scales were inspired by [34]. Based on the Common International Classification of Ecosystem Services [41], two categories of services were considered: the provisioning service of food for human nutrition (_food); and regulation and maintenance services referring to ecological resources and cycles (_ecol).

A second sphere, consisting of four questions, dealt with opinions about the future scenario for a sustainable supply of seafood. Sustainable seafood was defined as seafood that is either caught or farmed in ways that consider the long-term vitality of harvested species, the well-being of the oceans, and the livelihoods of people who depend on fishing activities [38]. Based on the European Commission [42], in a first question, respondents were asked their beliefs about the problem of scarcity in fish availability within the next ten years; in addition, two questions referred to opinions about those sustainable methods that do not deprive future generations of their benefits, by asking how more sustainable food could be obtained from harvesting the wild populations and from farming. As for future perspectives about fish scarcity (Fish_Scarcity scenario), response options were recoded as follows: a positive response (“Yes”) was given a score of 1; other responses (“Yes, but fish products from our sea can be replaced with those from another part of the ocean”; “No, fish species reconstitute themselves”; “No, it will happen in the long run”) were given a score of 2; “I do not know” answers were recoded with a score of 3. Opinions about how to make the fishing supply more sustainable (Fishing_Sust supply) were recoded as follows: solutions that referred to “creating marine environmental protection zones where fish species can reproduce’’ or to “catching new species of fish or shellfish that are currently unexploited or only marginally exploited” were assigned a score of 1; those suggesting “increasing the number of fishing vessels or using better gear for catching” or “enlarging the catching area” took a score of 2; finally, “I do not know” answers were assigned a score of 3. As for opinions about how to make farmed production more sustainable (Farming_Sust supply), responses were recoded as follows: solutions referring to “creating marine environmental protection zones where fish species can reproduce’’ or to “raising other species down the food chain that are not currently produced” were assigned a score of 1; “increasing the extension of the farming areas or the number of farms’’ took a score of 2; finally, “I do not know” answers were assigned a score of 3. Finally, a fourth question collected opinions about whether (Yes/No) respondents considered fishing (ME econ function_fishery) and farming (ME econ function_farming) among the most relevant activities of the blue economy within the next ten years.

The third section of the questionnaire was composed of five questions that dealt with people’s demand for fish products. At first, respondents were asked about their consumption frequency of selected fish products. The selection of fish products was made on the basis of a triple order of criteria with environmental implications: first, on the basis of the origin of the product (local or imported), of the food cultures to which they belong (Mediterranean, Northern Europe, Asia), and the source of captured or farmed fish; second, on the basis of different types of product (whether fresh, frozen, canned, smoked, transformed); and finally, on the basis of the most consumed fish in Italy [43]. To meet the above criteria, five products were considered: anchovies (local, Mediterranean, wild, fresh fish); cod filet; canned tuna fish; smoked salmon; and sushi, sashimi, and poke (imported, Asian, wild or farmed, transformed). In a second question, some nutritional knowledge related to seafood was investigated among students by considering their knowledge of the recommended weekly intake of seafood (Fish Consumpt_Intake). In two questions, students were asked to select from a list the least present element (carbohydrates) in the composition of fish (Fish_Absent component), and to declare their knowledge about safety risks in fish consumption (Fish Consumpt_Safety Risks) due to the presence of contaminants. Finally, the fifth question asked respondents’ opinions about the best ways to make the demand for seafood more sustainable within the next ten years (Fish_Sust demand): in addition to the options related to the reduction of human fish consumption and of non-food uses, other ways referred to a diversification of the demand toward novel and discarded seafood products.

The last section of the questionnaire collected individual characteristics, such as gender and area of residence (coastal, inland).

### 3.2. Methods

Descriptive statistics provide an overall summary of the survey’s responses over the investigated phenomena, useful to answer RQ.1.

The level of significance of differences in the distribution of responses to several variables by socio-demographic characteristics was examined. The non-parametric Mann–Whitney U test was used to compare two independent groups, and the Kruskal–Wallis test for more groups. All statistical tests were performed considering the significance levels of α = 0.05 and α = 0.01. The Mann–Whitney U test and the Kruskal–Wallis test are non-parametric alternatives to the *t* test and the one-way ANOVA, respectively, for two or more independent samples. The prerequisite of using a parametric analysis is that the data tested assume a normal (Gaussian) distribution, but this hypothesis could be questionable; for this reason, non-parametric tests are preferred to the parametric ones because they do not require a normal assumption. In addition, unlike the *t* test which compares mean values between two groups, the Mann–Whitney U test compares their median and is more appropriate with nominal or ordinal variables [20].

Finally, to answer RQ.2, a cluster analysis was performed based on variables catching respondents’ sensitivity, conflict awareness, opinions about future fish scarcity scenarios, and about alternatives for sustainable fish supply and demand. Cluster analysis is a statistical technique frequently used in research aimed at detecting homogeneous segments of consumers based on their sustainable food consumption [20,44,45]. Hierarchical methods are often selected within the family of clustering methods for several advantages, among which are that they do not require pre-specification of the number of clusters and are not sensitive to initialization conditions or to the order of the dataset [46,47]. The Euclidean distance was used as a measure to assess the pairwise differences among statistical units. A hierarchical cluster analysis was applied with Ward’s method, which is the only one among the agglomerative clustering methods that is based on a sum-of-squares criterion, producing groups that minimize within-group dispersion at each binary fusion [47]. Cluster analysis was performed by using the statistical software Stata/SE 13.1. The choice of the optimal number of clusters was based on the most used empirical elbow method: for different numbers of clusters, the objective functions are as follows:

$$R_{\left(k\right)}^{2}=\frac{B_{\left(k\right)}}{T}$$

where $B_{\left(k\right)}$ is the between-cluster variance for a number of clusters *k*. Reporting on a plot the number of clusters *k* and the $R_{\left(k\right)}^{2}$ values, a good choice for the number of clusters is *k* at which the graph shows an elbow. In addition, per each cluster solution, the Calinski–Harabasz pseudo-F and the Duda–Hart Je(2)/Je(1) indexes available for hierarchical clustering were considered as a further stopping rule for detecting the number of clusters. For both rules, larger values indicate more distinct clustering.

## 4. Results

### 4.1. Descriptive Statistics

The final sample was composed of 778 individuals, of which 56% were female, 65% lived in inland areas and 70% in the bigger cities. Students with food allergies or who did not consume seafood dropped out of the survey.

Respondents revealed moderate to high sensitivity to the environmental status of marine ecosystems, and almost 77% of the sample declared that they were interested. As for the awareness and concern about the existence of conflicts between ecosystem conservation goals and economic activities, around two-thirds of students agreed, although a non-negligible percentage of individuals (20%) were irresolute.

Among the most important values assigned to the marine ecosystems, the provisioning of food for human nutrition was identified by 56% of respondents, but the ecological value was considered even more important (61%); 30% of respondents selected both the provisioning and the ecological values, while 14% of respondents did not select either of the two options.

Respondents paid more attention to the ecological value than to the economic one also in relation to threats: 77% of the sample considered that the ecological value could suffer future threats, while half of the sample considered the economic value to be at risk; both values were selected by 36% of respondents, while 9% did not select either value. As for the importance of the seafood industry in the future blue economy, fishery was considered more important (43%) than mariculture (35%), and 35% of students selected neither.

People’s assessment of a future phenomenon of fish scarcity gave the following distribution of answers: 58% of respondents thought that captures are limited and rapidly declining all over the world; 22% of them did not have any idea; the remainder of the sample were of the opinion that the problem will exist but could be solved by catching elsewhere in other oceans (11%), or that it will not exist because fish species reconstitute themselves (5%), or it will happen in the long run (4%).

As regards the actions useful to sustainably increase fish supply, the irresolute answers were not as high as expected (about 15%). The most agreed solution is to create a marine protected area to benefit capture and farmed fish populations through nursery and spillover of individuals from the protected area (about 80%). Secondly, people considered the market exploitation of new capture and farmed species that are marginally or not at all exploited or produced (about 40%). Thirdly, as regards the wild source, young people gave similar importance to the two less sustainable options for increasing the supply of fish products, i.e., by enlarging the fishing areas and by increasing the fishing effort (about 30%); as regards the farmed source, increasing the production volume, or the size of breeding areas and facilities followed the ranking of people’s opinions on how to sustainably increase the reared production (about 35%).

Opinions about the solutions that may make fish demand more sustainable in future scenarios put first the reduction of wild caught fish used to produce meal, oil, or other non-foods (63%). Secondly, more than half of the sample considered the increase in consumption of new or not commercially viable species as an opportunity to make the demand more sustainable. A quite high percentage of people (48%) considered the reduction of per capita fish consumption as an option for making more sustainable the seafood demand, although the Italian Dietary Guidelines recommend an increase in fish intake [48]; finally, 37% of the sample were in favor of consuming novel seafood for human nutrition, such as algae and jellyfish.

Anyway, Gen-Z declared themselves to be informed about the recommended fish intake: 15% of respondents did not answer, 69% stated the intake should be 2–3 servings, 12% selected “once a week”, and less than 4% answered four servings or more per week. As for the least frequent component of seafood, 61% of students properly selected carbohydrates, while the remainder of the sample gave the wrong answer.

The consumption frequency of selected seafood (Table 1) reveals that canned tuna is the most consumed product on a weekly basis, followed by smoked salmon and cod filet. At the opposite end of the scale, students declared they consumed anchovies and Japanese seafood with a frequency of once a month, the first fish being caught in every Italian sea, the second product being usually made with imported fish ingredients.

Regarding students’ knowledge about fish contamination, responses converged the most on microplastics (79%), biological contaminants (74%), and metals (72%), while pathogenic parasites and microorganisms were less known (around half of the sample); all in all, the level of knowledge about these risks seems quite high, also considering the percentage of students declaring their knowledge of more than three types of contaminants (47%).

### 4.2. Test Analysis

Table 2 reports the results of the test analysis, highlighting the variables with significant differences by demographics. At a global glance, it emerged that both the gender and the area of residence were differentiated over several variables.

People’s awareness of potential conflicts between the preservation of the marine environment and economic activities differs by gender, with females showing more awareness than males. In addition, the level of perceived future importance of the ecological value related to marine environments is significantly different by gender, with females more frequently selecting this value than males. Students’ knowledge about some risks associated with food consumption differs by gender, with males having more knowledge than females. No differences emerged in relation to the perception of risk from microplastics and metals by gender and area of residence.

The area of residence makes a significant difference to the sensitivity about the environmental status of the sea. Unexpectedly, those who live inland have greater sensitivity than coastal residents; this difference is confirmed for the awareness related to conflicts between economic activities and conservation of the marine environment. In contrast, the perceptions of the future impacts the ecological value will suffer in the future, as well as the role of farming activities in the development of the blue economy, are higher among coastal residents than among students living in inland areas. No differences emerged regarding the importance of the ecological value and the food-provisioning marine ecosystem services.

### 4.3. Cluster Analysis

In order to answer RQ.2, five clusters were detected in the sample. Table 3 reports the mean score of the variables included in the analysis. Concerning RQ.3, Table 4 shows the frequency of fish consumption and socio-demographic statistics among the groups.

The first cluster consists of about 20% of the individuals. These students declared being not so much interested in the environmental status of marine environments and in the existence of conflicts between marine conservation goals and economic uses of marine resources. As regards the ways for increasing the fish supply from fishing or farming sources, they tended to select only one right option, the second response being wrong or irresolute; a similar result concerns the question related to the ways for making seafood more sustainable. For the questions related to the importance of and the threats to marine values, they responded by selecting a single option—the ecological value (24%) or the economic one (36%)—and only 25% selected both values. The provisioning of seafood from both wild and farmed sources was among the most important economic activities they envisaged for the future blue economy. They did not consider so much the safety issues in the consumption of fish and gave wrong answers about the presence of carbohydrates in the nutritional composition of fish. These students reported the lowest frequency of fish consumption for all seafood and any single product except Asian seafood; finally, as for the consumption of cod, which emerges as the only product whose consumption significantly differs among the clusters, this group declared a very low frequency of consumption compared with other groups. Students are equally distributed by gender; for this characteristic, this group is different from the whole sample and the other clusters. The distribution of people by place of residence includes a high percentage of individuals living in coastal areas, differently from the characterization of other groups.

The second cluster contains 23% of students in the sample. Differently from the previous profile, individuals in the group declared a high interest in the environmental status of the marine ecosystem. Nevertheless, they did not give a high weight to conflicts and to the problem of fish scarcity. On the other hand, they gave right answers regarding the options for intervening on the supply and the demand side to make fishing and farming production more sustainable. As for the prospective value of marine environments, this group assigned higher importance to the provisioning value (34%) than the ecological value (23%), but another third of people did not recognize any of these values. These students are quite conscious of the threats to the ecological value (47%), more than to the economic functions of marine ecosystems (18%). Concerning the economic importance attached to catching rather than farming, this group is quite peculiar: the highest percentage of people (58%) believe neither fishing nor mariculture will be the most important activities in the future marine economy, but 28% of individuals attach a greater importance to mariculture than fishing (only 10%). On average, this group declared the highest frequency of consumption of fish products overall and the highest of Asian seafood. Compared with other clusters, this group mainly includes people living in inland areas.

The third cluster is the smallest one, including 14% of individuals. This cluster accounts for the highest percentage of individuals—almost 90%—convinced that in the future there will be a problem of fish scarcity for satisfying human needs; as in the following cluster, this group strongly agrees on the existence of conflicts between conservation goals and economic activities. Furthermore, more than half of the members are sensitive to the environmental status of marine ecosystems. In addition, the right answers prevailed among opinions about sustainable alternatives on the supply and demand sides, while wrong answers were very few compared with other groups. The topic of values characterizes the cluster: more than half of the students assigned importance to both ecological and food-provisioning values of marine areas, but 35% of them attached importance to the ecological value, while a lower percentage of students considered the economic function. This profile is consistent with opinions related to the values threatened in future scenarios, where both ecological and fish-provisioning services were often jointly selected, but the ecological option convinced more than 90% of students in the group. The cluster is strongly oriented toward recognizing the importance of fishing activity (60%) rather than farming (15%); a high percentage (22%) of individuals gave importance jointly to fishing and farming compared with those (3%) denying the importance of both activities. This group declared the highest consumption of canned tuna and the lowest of smoked salmon. Females are more numerous than males, and location in inland areas is higher than in other groups.

The fourth cluster includes 18% of students. It is quite similar to the previous group in terms of high awareness about a scarcity scenario (86% of people). In contrast, these students were not so accurate in choosing the proper way for meeting sustainable fish demand (52% selected two wrong options). Regarding the importance of values, this group favored the ecological value (42%), but there were also a lot of people (43%) who selected both ecological and food-provisioning values. The distribution of responses about impacts is quite peculiar: the prevailing opinion (44%) is that both ecological and economic spheres will be threatened and only 15% of individuals denied impacts on both values. As for the food-provisioning activity in future scenarios, people were more oriented towards giving importance to farming (25% of responses compared with 19% to fishing), but 52% of students believed that neither activity is important. These students declared the lowest frequency of consumption of canned tuna. As for the socio-demographic characteristics, this is the most feminine group among the clusters.

The fifth cluster is the biggest one, with 26% of students. A first aspect characterizing the group is the uncertainty of 83% of people about the future problem of fish scarcity; another 10% consider that the problem will emerge in the long term. Responses about solutions to make more sustainable the caught and farmed productions reveal a quite high selection of wrong options. As for the importance of values attached to marine ecosystems, the distribution of students is quite balanced between those indicating the ecological value (32%), the food-provisioning one (28%), or both values (28%). In contrast, threats were mainly assigned to the ecological sphere (41%) or jointly with the economic sphere (37%). Opinions about the most important economic activities in the future exploitation of marine resources did not differ so much as in the previous group, but here were more oriented toward fishing (31% of responses) than farming (21%). This group reported the lowest frequency of consumption of Asian seafood, while it has a profile similar to that of the entire sample for other seafood products. A high percentage of people living in inland areas is observed in the cluster.

## 5. Discussion

Differences in study aims, methods, and population targets do not allow a direct comparison of the present findings with those from prior studies that, as reported in the literature section, addressed more the sustainability of food in general than that of seafood products, and were mainly focused on university students instead of younger people [35,36,49,50]. Furthermore, specific investigation of people’ beliefs about the future sustainability of seafood supply and demand is not so common in the literature [51] that has often investigated current sustainable food choices.

As regards Italy, there are studies that show that Italians are increasingly informed and aware about environmental sustainability issues that influence their consumption choices [52]. It has been shown that preferences for products with environmental sustainability labels increase when consumers have adequate knowledge of what the labels mean [53]. As regards marine environmental consciousness and concern, recent studies have shown that Italians are very aware and concerned about marine plastic pollution, its impacts, and solutions [54], as well as about the sustainability challenges related to seafood consumption [20,55].

Differently from our findings, a gender effect did not emerge in the segmentation study by Sánchez-Bravo et al. [45]. Elsewhere, being a woman was reported as a factor related to sustainable food consumption [34,56]. In contrast, Pocol et al. [57] analyzed types of eating behaviors—a priori defined as sustainable vs. unsustainable—among university students in Romania, Bulgaria, and Moldova and found differences based on gender and urban/rural residence.

The knowledge that our sample reveals about the nutritional intake and potential safety risks of seafood consumption is quite in line with the findings emerged among Millennial Turkish consumers [35]. In contrast, Yüksel and Önal [58] highlighted a significant lack of knowledge among Turkish university students regarding the characteristics of sustainable nutrition, with a gender effect related to male students mostly thinking that foods have no effect on the environment. In line with our findings are results of Anuar et al.’s [36] study on Malaysians’ willingness to purchase unfamiliar seafood products if they were informed about that being the most sustainable option.

Among the ways to make future supply and demand for wild-caught and farmed products environmentally sustainable, our respondents placed importance on the catching and raising of new or discarded species, including unharvested or marginally exploited ones. This finding is quite interesting and promising as it is an expression of the growing environmental awareness among Italian young people, which can contribute to the reduction of the environmental pressures of seafood demand on fish stocks. This is also very important from the perspective of both the UN SDGs 12 and 14. Moreover, this result can support the intensification of policy actions and tools useful for further developing environmental awareness among young people, tools among which those aimed at increasing literacy, knowledge, and education of young people are of particular importance. In this regard, it is comforting that this result does not emerge only in the Italian case. In fact, these study results fit well with those reported in the studies by Anuar et al. [36] and Davis [38], where U.S. university students wanted to be more engaged with the consumption of underutilized species although they were highly unaware of and unfamiliar with local and underutilized species. As regards the local provenance of fish species (in this study, anchovies), our Gen Z seems unaware or may not like local fish.

The profiles of our clusters share some similarities with the segments detected by Bollani et al. [59] dealing with the topic of ecological sustainability in the food sector within a sample of university students in three Italian cities. In particular, regarding respondents’ awareness of the definition of sustainability, similarities could be found between our sensitive groups and the so-called segments of “info-supporter” and “proactive-oriented” in the study by Bollani; there are also similarities between our insensitive and unaware students and Bollani’s “indifferent” segment.

Our segments are somewhat in line with the groups obtained by Su et al. [39]: Italian and U.S. Gen Zs are becoming increasingly concerned about the environment; the sensitive groups in this study share some general characteristics with the three U.S. consumer groups classified as sustainable activists, believers, and moderately conscious; furthermore, similar results concern the role of gender in significantly differentiating groups, with women more involved in the most sustainable groups.

When the sustainability characteristics of the segments are linked to the frequency of consumption of the seafood products considered in the study, a nexus does not emerge in terms of a greater frequency of consumption of all products and of the local one (anchovy). This result is in line with those of studies showing that people who consider themselves environmentally conscious do not necessarily have a positive attitude toward fish [60] and pay attention to environmental attributes of seafood [21,28].

## 6. Conclusions

The study findings provide insights into the opinions of the Italian seafood consumers of today and tomorrow.

Indeed, the focus on Gen Z’s approach toward the sustainability of seafood arises from the belief that an in-depth knowledge of the opinions of today’s young people can reveal future tendencies about the preservation of marine resources and the seafood provisioning service. Furthermore, young people’s approaches towards the environmental sustainability of seafood production and consumption are relevant because, at a young age, individuals begin to develop certain consumption patterns that can have long-term effects on the sustainability of the seafood market, its supply, and demand. Under both perspectives, Generation Z may support the implementation of SDG 14 by contributing to the conservation of life under water and the sustainable use of marine resources for human consumption, as well as of SDG 12 by adopting sustainable seafood consumption choices that might drive responsible production practices.

The segmentation analysis highlights the different sustainability profiles of each group of young people and seafood consumers. Several groups showed profiles of sensitivity and awareness about the investigated topics, but two groups comprising almost half of the sample were insensitive, unaware, or irresolute about the sustainability of seafood production and consumption. Furthermore, environmental awareness and sensitivity among groups do not appear to be strongly related to the frequency of consumption of the most sustainable seafood species among those considered.

The most agreed-upon solution for the sustainability of future seafood supply is considered to be the creation of marine protected areas for benefiting capture and farmed fish populations through nurseries and the spillover of individuals from protected seas. This opinion is promising in the light of the EU Biodiversity Strategy and supports the national target of at least 30% of seas being under protection by 2030.

Sustainable marine policy as well as seafood policy should ideally focus on groups that score highly in terms of environmental profile. Furthermore, actions should also be oriented towards promoting awareness among groups that show lower consciousness, higher misperception, or declare irresolute opinions. Policy makers and industries should develop educational and marketing strategies tailored to the various groups and provide meaningful and accurate information on the environmental sustainability of fish production and consumption.

Some limitations of the study could be related to the characteristics of the sample that, despite being large and quite homogeneous in age (18–20 years), does not cover the full range of individuals belonging to Generation Z. In order to obtain a wider perspective, the study could be replicated in other countries and broaden the investigation into other seafood species that young individuals could know, consume, and appreciate.

## Figures and Tables

**Table 1:** Consumption frequency of seafood products (% of respondents).

	Once a Month	2/3 Servings a Month	Once a Week	2/3 Servings a Week	More than 3 Servings a Week
Anchovy	40.2	27.6	19.0	10.3	2.9
Cod filet	23.9	26.6	24.4	18.4	6.7
Canned tuna	13.4	16.4	24.6	28.5	17.1
Smoked salmon	17.3	22.8	27.2	23.9	8.8
Sushi, sashimi, poke	40.6	35.2	14.3	7.1	2.8

**Table 2:** Differences in the distribution of phenomena by socio-economic characteristics.

	Gender	Area of Residence
Sensitivity		* (I)
Marine protection conflicts	* (F)	** (I)
ME_importance_ecol		
ME_importance_food		
ME_menace_ecol		** (C)
ME_menace_econ	** (F)	
ME_econ function_fishery	* (M)	
ME_econ function_farming		* (C)
Farming_Sust supply	** (M)	
Fish scarcity scenario	** (F)	
Fish Consumpt_Anchovies	** (M)	
Fish Consumpt_Cod		* (I)
Fish Consumpt_Intake	** (F)	
Fish Consumpt Safety Risks_Parasites	* (M)	
Fish Consumpt Safety Risks_Microorganism	* (M)	
Fish Consumpt_Safety Risks_Contaminants	* (M)	
Fish_Absent Component		** (I)

Legend: * α = 0.10; ** α = 0.05.

**Table 3:** Clusters’ profiles.

Cluster Label Variables	1. Gen Z Insensitive and Unaware	2. Gen Z Sensitive and Informed	3. Gen Z Sensitive, Value-oriented, and Aware	4. Gen Z Sensitive, Worried, but Unaware	5. Gen Z Uninformed and Irresolute	Total Sample
Cluster size (N)	152	177	108	140	201	778
%	19.5	22.8	13.9	18.0	25.8	100.0
Sensitivity ^(a)^	3.243	4.277	4.389	4.064	3.836	3.938
Marine protection conflicts ^(a)^	3.197	3.463	4.231	4.100	3.662	3.684
ME_importance_ecol+food ^(b)^	2.408	2.876	1.537	1.736	2.234	2.228
ME_menace_ecol+food ^(b)^	2.217	1.904	1.685	1.993	1.935	1.959
Fishing_Sust supply_score ^(c)^	2.211	1.718	1.648	2.136	2.149	1.991
Farming_Sust supply_score ^(c)^	2.428	1.719	1.657	2.036	2.358	2.071
Fish_Sust demand_score ^(c)^	2.125	1.915	1.898	2.500	2.114	2.111
Fish scarcity scenario ^(d)^	1.362	1.232	1.111	1.143	2.826	1.636
Fishing/Farming_Importance ^(e)^	2.184	3.401	1.981	3.250	2.816	2.788
Fish Consumpt_Intake ^(f)^	2.546	2.695	2.750	2.657	2.512	2.620
Fish Consumpt Safety Risks ^(g)^	2.158	2.350	2.407	2.314	2.119	2.254
Fish_Absent Component ^(h)^	1.428	1.424	1.287	1.386	1.413	1.396
Average FFQ ^(i)^	2.460	2.635	2.509	2.521	2.513	2.536

Legend: (a) from 1 = totally disagree to 5 = totally agree; (b) 1 = “Yes” both values; 2 = “Yes” one of the values, 3 = “No” both values; (c) 1 = all appropriate answers; 2 = at least one appropriate answer: 3 = inappropriate answers; (d) 1 = appropriate answer; 2 = inappropriate answer; 3 = do not know; (e) 1 = fishing and farming; 2 = only fishing; 3 = only farming; 4 = neither; (f) 1 = once a week; 2 = two–three times a week; 3 = four times or more; (g) 1 = fewer than three risks; 2 = three risks; 3 = more than three risks; (h) 1 = right answer; 2 = wrong answer; (i) 1 = once a month, 2 = two/three servings a month, 3 = once a week, 4 = two/three servings a week, 5 = more than three servings a week.

**Table 4:** Frequency of fish consumption and socio-demographics by clusters.

Cluster Label Variables	1. Gen Z Insensitive and Unaware	2. Gen Z Sensitive and Informed	3. Gen Z Sensitive, Value-Oriented, and Aware	4. Gen Z Sensitive, Worried, but Unaware	5. Gen Z Uninformed and Irresolute	Total Sample
Seafood consumption frequency						
Anchovy	2.02	2.13	2.05	2.06	2.10	2.08
Cod filet	2.36	2.73	2.56	2.61	2.58	2.57
Canned tuna	3.13	3.25	3.29	3.08	3.23	3.20
Smoked salmon	2.78	2.97	2.77	2.85	2.80	2.84
Sushi, sashimi, poke	2.01	2.07	1.92	1.99	1.83	1.96
Gender						
Female	50.0%	56.5%	59.3%	66.4%	52.7%	56.4%
Male	50.0%	43.5%	40.7%	33.6%	47.3%	43.6%
Area of residence						
Coastal	42.1%	32.2%	31.5%	35.7%	32.3%	34.7%
Inland	57.9%	67.8%	68.5%	64.3%	67.7%	65.3%

## Data Availability

The data used to support the findings of this study can be made available by the corresponding author upon request.
